# A Perilipin Gene from *Clonostachys rosea* f. *Catenulata* HL-1-1 Is Related to Sclerotial Parasitism

**DOI:** 10.3390/ijms16035347

**Published:** 2015-03-09

**Authors:** Zhan-Bin Sun, Shi-Dong Li, Zeng-Ming Zhong, Man-Hong Sun

**Affiliations:** Key Laboratory of Integrated Pest Management in Crops, Ministry of Agriculture, Institute of Plant Protection, Chinese Academy of Agricultural Sciences, Beijing 100081, China; E-Mails: twins5616@126.com (Z.-B.S.); lisd@ieda.org.cn (S.-D.L.); zhongzengming@163.com (Z.-M.Z.)

**Keywords:** *clonostachys rosea* f. *catenulate*, *Sclerotinia sclerotiorum*, sclerotia, perilipin, mycoparasitism, ESTs

## Abstract

*Clonostachys rosea* f. *catenulata* is a promising biocontrol agent against many fungal plant pathogens. To identify mycoparasitism-related genes from *C. rosea* f. *catenulata*, a suppression subtractive hybridization (SSH) cDNA library of *C. rosea* f. *catenulata* HL-1-1 that parasitizes the sclerotia of *S. sclerotiorum* was constructed. 502 clones were sequenced randomly, and thereby 472 expressed sequence tags (ESTs) were identified. Forty-three unigenes were annotated and exhibited similarity to a wide diversity of genes. Quantitative real -time PCR showed that a perilipin-like protein encoding gene, *Per3*, was up-regulated by 6.6-fold over the control at 96 h under the induction of sclerotia. The full-length sequence of *Per3* was obtained via 5' and 3' rapid identification of cDNA ends. Overexpression of *Per3* in HL-1-1 significantly enhanced the parasitic ability on sclerotia. The results indicated that *Per3* might be involved in the mycoparasitism of *C. rosea* f. *catenulata* HL-1-1. This is the first report of a perilipin as a potential biocontrol gene in mycoparasites. The study provides usefu l insights into the interaction between *C. rosea* f. *catenulata* and fungal plant pathogens.

## 1. Introduction

*Clonostachys rosea* f. *catenulate* (syn. *Gliocladium catenulatum*) is a mycoparasite capable of antagonizing a range of fungal plant pathogens, such as *Sclerotinia sclerotiorum*, *Rhizoctonia solani*, *Fusarium* spp., and *Pythium* spp. [1,2,3,4]. It has been widely used as a biological control agent [5,6,7,8]. Mycoparasitism is an important characteristic of biological control agents; the mechanism of mycoparasitism was initially studied in a few mycoparasite—Fungus interactions. Steindorff *et al.* identified various genes encoding transporters and hydrolytic enzymes that were differentially expressed in *Trichoderma harzianum* TR274 growing on *S. sclerotiorum* cell walls compared with the glucose control [9]. However, the specific functions of these genes were not studied. Thus, screening key biocontrol-related genes and studying their functions to understand biocontrol mechanisms will contribute to improving the efficiency of biocontrol agents.

A variety of genes have been confirmed as associated with biological control of plant pathogens. In comparison with the wild strain, a transformant of *Metarhizium anisopliae* carrying a neurotoxin gene *AalT* from *Androctonus australis* exhibited a higher fungal toxicity to *Manduca sexta* and *Aedes aegypti*, and stronger pathogenicity against *Hypothenemus hampei* [10,11]. Overexpression of the *Trichoderma harzianum erg1* gene (involved in the synthesis of ergosterol) was suggested to improve the biocontrol ability of the *Trichoderma* strains and their interaction with plants [12]. Kosawang *et al.* [13] disrupted a zearalenone lactonohydrolase encoding gene, *zhd101*, from *C. rosea*, which resulted in weak control of a zearalenone-production fungus *Fusarium graminearum*. Dubey *et al.* [14] identified an ATP-binding cassette transporter encoding gene *abcG5*, disruption of which failed to protect barley seedlings from *F. graminearium* foot rot disease. It was also suggested that knockdown of a virulence related serine protease gene *pacC* attenuated the virulence of *C. rosea* to nematodes [15].

Perilipin is a lipid droplet protein that regulates fat storage and lipid homeostasis in organisms by responding to signals that stimulate triacylglycerol-degradation [16,17]. It can bind to the surface of lipid droplets of adipocytes and restrict the access of lipases [18]. Previous studies on the function of perilipins mainly focused on its role in cellular lipid metabolism in mammals. Tansey *et al.* found that a *perilipin* null mouse had less fat and a higher metabolic rate, and was resistant to diet-induced obesity, compared with normal mice [19]. The role of perilipin has also been studied in Drosophila, where deletion of a perilipin encoding gene, *Lsd2*, decreased the level of neutral lipids [20]. Although perilipin has been identified from fungal species, limited research on its function has been conducted. A perilipin homolog, MPL1, from *M. anisopliae* was reported to regulate lipid metabolism, appressorial turgor pressure and virulence. The *Mpl1* mutant of M. anisopliae had thinner hyphae, fewer lipid droplets (particularly in the appressoria), and a decrease in total lipids [21]. This was the first report to suggest that perilipin is a virulence determinant in insect biocontrol agents. However, little is known about the function of perilipin in mycoparasitic fungi.

*C. rosea* f. *catenulata* strain HL-1-1 was originally obtained via sclerotia baiting, and showed great potency to manage a range of fungal plant pathogens [22]. To identify mycoparasitism-related genes, a suppression subtractive hybridization (SSH) library of HL-1-1 colonizing the sclerotia of *S. sclerotiorum* was constructed [23]. In this study, 502 clones from the cDNA library were sequenced and analyzed, among which a perilipin-like protein coding gene, named *Per3*, was detected. The expression of *Per3* under the induction of sclerotia was remarkably up-regulated, and the parasitic ability of mutants overexpressing *Per3* was enhanced significantly, suggesting that perilipin might be involved in mycoparasitism of *C. rosea* f. *catenulata*.

## 2. Results

### 2.1. Sequence Analysis of ESTs

502 clones from the SSH cDNA library of *C. rosea* f. *catenulata* HL-1-1 were sequenced, among which 472 high quality expressed sequence tags (ESTs) were identified. These ESTs were classified into 232 unigenes, including 35 contigs and 197 singletons. Of the 232 unigenes, 136 (58.6%) showed similarity to genes with unknown functions and 53 (22.9%) showed no similarity to any sequences in the databases and were considered as fragments of novel genes. Forty-three unigenes (18.5%) displayed sequence similarity (*E*-value < 10^−5^) to entries in the GenBank non-redundant protein database, exhibiting similarity to a wide range of genes involved in metabolism (e.g., aldehyde dehydrogenase, chitin synthase and fatty acid oxygenase), energy (e.g., ubiquinone oxidoreductase and ATP synthase), protein fate (e.g., ribosomal proteins and heat shock proteins), transcription (e.g., transcription factors and endonucleases), cellular transport (e.g., sodium phosphate and major facilitator superfamily (MFS) transporter), and cell rescue and defense (cytochrome P450) (Table 1). Part of the unigenes was annotated by Blast2GO software (Supplementary Table S1). Among these functional unigenes, some were related to the biocontrol process, including cell wall degrading enzyme endoglucanase [24,25], cytochrome P450 [26,27], and ubiquitin [28]. A unigene was identified that was very similar to a perilipin-like protein, which was reported as an important virulence determinant in the insect biocontrol agent *M. anisopliae* [21]. This unigene was named *Per3*. We determined the expression of *Per3* in different parasitic stages and cloned the full-length gene from HL-1-1.

**Table 1:** Functional analysis of differentially expressed genes in *C. rosea* f. *catenulata* HL-1-1 parasitizing the sclerotia of *S. sclerotiorum* by BlastX.

Gene	Accession Number	Organism	Blast Homology Search	**E**-Value
*Per3*	ABI18161	*Metarhizium anisopliae*	Perilipin-like protein	4e^−44^
1-31	EIW51416	*Trametes versicolor*	CHK1 checkpoint-like protein	4e^−16^
2-9	EEY19892	*Verticillium alfalfae*	Endoglucanase	4e^−26^
2-13	XP_002294430	*Thalassiosira pseudonana*	rRNA intron-encoded homing endonuclease	9e^−23^
2-25	EPE03816	*Ophiostoma piceae*	Sodium phosphate	1e^−34^
2-26	XP_001891758	*Brugia malayi*	Transcription factor	2e^−23^
2-27	CCF47281	*Colletotrichum higginsianum*	Shwachman-Bodian-Diamond syndrome protein	2e^−35^
2-29	NP_690845	*Saccharomyces cerevisiae*	Tar1p	1e^−23^
2-49	XP_751750	*Aspergillus fumigatus*	Fatty acid oxygenase PpoA	2e^−41^
2-84	XP_002567725	*Penicillium chrysogenum*	Pc21g06830	1e^−36^
2-90	ELA35160	*Colletotrichum gloeosporioides*	Trichothecene c-15 hydroxylase	7e^−39^
3-4	XP_003844958	*Leptosphaeria maculans*	Similar to polyketide synthase	2e^−33^
4-33	EJP62467	*Beauveria bassiana*	4-hydroxyphenylpyruvate dioxygenase	5e^−56^
4-37	1101405A	*Saccharomyces cerevisiae*	Ubiquitin precursor	5e^−86^
4-47	EFY90398	*Metarhizium acridum*	ADP,ATP carrier protein	6e^−23^
4-49	EQK98455	*Ophiocordyceps sinensis*	Glucose-repressible protein	6e^−26^
5-35	EGC42647	*Ajellomyces capsulatus*	Transcript antisense to ribosomal RNA protein	5e^−17^
5-84	EON96835	*Togninia minima*	Heat shock protein 30	1e^−21^
6-9	EJP67708	*Beauveria bassiana*	Translationally controlled tumor protein-like variant I	3e^−38^
6-35	EHK46444	*Trichoderma atroviride*	Plasma membrane ATPase	1e^−75^
7-13	EFQ32165	*Colletotrichum graminicola*	Cytochrome b561	2e^−14^
7-32	EGV64838	*Candida tenuis*	Ubiquitin	2e^−139^
7-52	EGS20521	*Chaetomium thermophilum*	Zinc finger domain-containing protein	2e^−5^
7-69	EKG14599	*Macrophomina phaseolina*	MFS transporter	1e^−39^
7-73	EQB58524	*Colletotrichum gloeosporioides*	Aldehyde dehydrogenase	2e^−24^
7-77	EKD20522	*Marssonina brunnea* f. sp. *multigermtubi*	Metallopeptidase family M24	5e^−9^
7-82	EFQ34155	*Glomerella graminicola*	Archaeal flagellin N-terminal-like domain-containing protein	2e^−5^
7-85	ABY21303	*Mytilus trossulus*	Cyp-like protein	2e^−13^
8-7	EGX92208	*Cordyceps militaris*	ATP synthase subunit E	2e^−15^
8-16	EJU02320	*Dacryopinax* sp.	Plant senescence-associated protein	2e^−63^
8-20	EGR52174	*Trichoderma reesei*	Glycoside hydrolase family 16	2e^−26^
8-21	CCT68298	*Fusarium fujikuroi*	Neutral amino acid permease	1e^−5^
8-22	EFY90747	*Metarhizium acridum*	NADH:ubiquinone oxidoreductase 18.4 kD subunit	8e^−36^
8-24	EGS18562	*Chaetomium thermophilum*	Chitin synthase-like protein	1e^−8^
8-25	BAA10929	*Nicotiana tabacum*	Cytochrome P450 like TBP	9e^−21^
8-48	EFY88095	*Metarhizium acridum*	Peptidoglycan binding domain containing protein	6e^−27^
8-70	XP_003655325	*Thielavia terrestris*	Histone H4-like protein	3e^−54^
8-76	EGY21577	*Verticillium dahliae*	Prohibitin-2	2e^−138^
8-89	XP_002384050	*Aspergillus flavus*	NADH-cytochrome B5 reductase	3e^−60^
9-25	EKD19306	*Marssonina brunnea* f. sp. *multigermtubi*	60S ribosomal protein L11	3e^−9^
9-58	WP_005077491	*Mycobacterium abscessus*	Dienelactone hydrolase	5e^−39^
9-78	XP_002474926	*Postia placenta*	Chloroperoxidase-like protein	3e^−5^

### 2.2. Quantitative Determination of Per3 Expression

**Figure 1:**
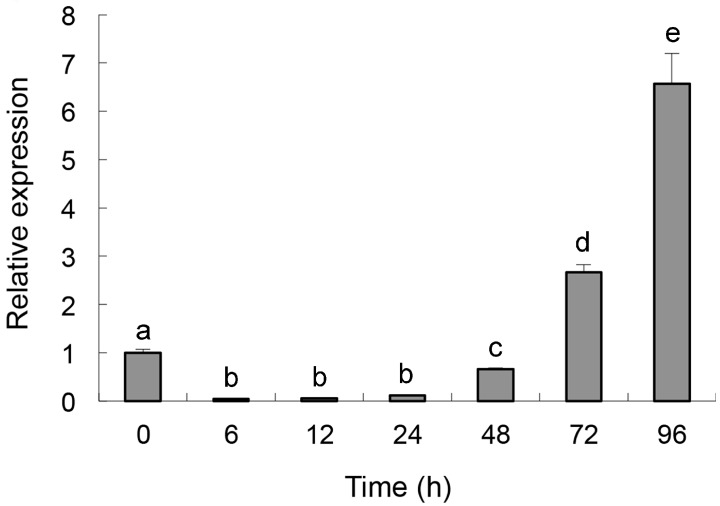
Expression of *Per3* in *C. rosea* f. *catenulata* HL-1-1 under the induction of sclerotia of *S. sclerotiorum*, using quantitative real-time PCR. Error bars indicate the standard deviation of three replicates. Different letters represent significant differences (*p* < 0.05) according to Duncan’s multiple range test.

The primer sets designed for *Per3* and those for the reference gene β*-tubulin* successfully amplified a single product with the expected size in all experiments (Supplementary Figure S1).The identity of each product was confirmed by sequencing. Real-time quantitative PCR showed that the relative expression level of *Per3* was down-regulated at the beginning of sclerotia induction (0–12 h). Subsequently, the expression of *Per3* was up-regulated, reaching its highest level (6.6-fold over the control) at 96 h (*p < 0.05*) (Figure 1).

### 2.3. Cloning and Characterization of Per3

*Per3* PCR products of 500 bp and a 750 bp were obtained by 3' and 5' rapid amplification of cDNA ends (RACE), respectively. After gene splicing and PCR verification, a full-length cDNA of 1231 bp was generated (Supplementary Figure S1). The complete genomic DNA sequence of *Per3* was amplified using the primer pair *Per3* F and *Per3* R. The PCR product comprised 1284 bp with a 53 bp intron at position 377–429 (GenBank accession number KF269990).

The full-length cDNA had a 555bp open reading frame (ORF) from position 314 (ATG) to 868 (TAA), encoding a putative protein of 184 amino acids. The predicted isoelectric point (IP) of the protein was 6.08 and theoretical molecular weight was 20.2 kDa. The amino acid sequence showed high homology to perilipin-like proteins in *M. anisopliae* (73.3%, ABI18161), *Cordyceps militaris* (62.4%, EGX91943) and *Beauveria bassiana* (60.1%, EJP62332). These comparisons indicated that Per3 contained a perilipin domain between amino acids 40 to 111. No signal peptide, transmembrane domains or typical hydrophobic domain was detected in Per3.

**Figure 2:**
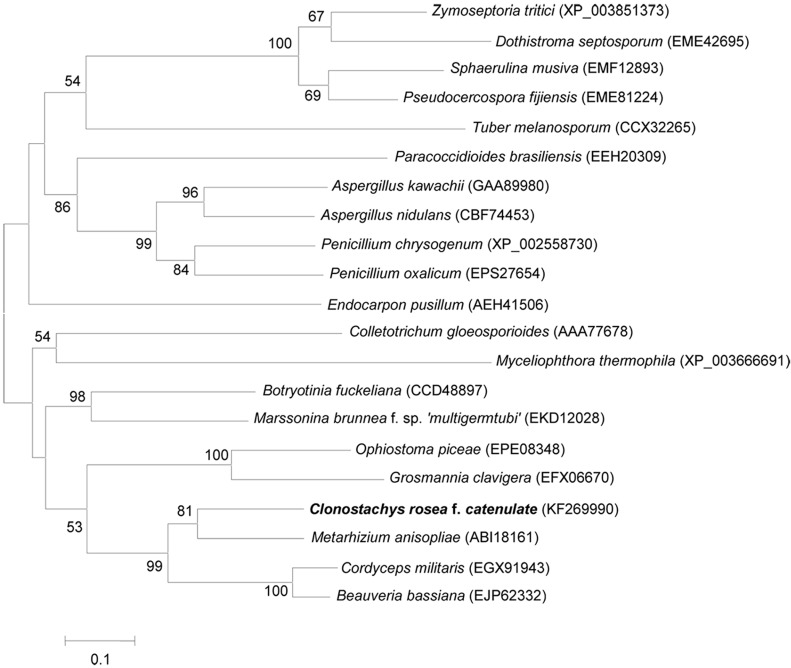
Neighbor-joining tree of protein Per3 from *C. rosea* f. *catenulata* HL-1-1 constructed using MEGA 5.1. Numbers in parentheses represent accession numbers in the NCBI sequence database. Numbers at the nodes indicate the bootstrap values on neighbor-joining analysis of 1000 bootstraps. Bars (=0.1) represent sequence divergence.

Twenty amino acid sequences with high similarity to Per3 were identified using BlastP and used to construct a neighbor-joining tree to investigate the phylogeny of perilipins (Figure 2). The tree showed that Per3 from *C. rosea* f. *catenulata* was most closely related to that of *M. anisopliae* (73.3%), demonstrating Per3 is a member of the perilipin family.

### 2.4. Overexpression of Per3 in C. rosea f. catenulate HL-1-1

The *Per3* gene sequence, together with *gpdA* and *trpC* fragments, was successfully integrated into the pAN7-1 vector, which was used to transform *C. rosea* f. *catenulate* HL-1-1. By stability verification, 320 transformants were obtained on hygromycin resistance plates. Phenotypic differences among the transformants were observed: their hyphae were either loose or dense, and the color of the colonies varied from gray to dark green. One hundred and two transformants were selected randomly and tested. There was no significant difference in the growth rate (*p* > 0.05), with colony diameters ranging from 6.02 to 7.35 cm in seven days. However, significant differences in sporulation were observed, with the highest level of 6.23 × 10^8^ spores·plate^−1^ and the lowest amount of 2.35 × 10^7^ spores·plate^−1^ (*p* < 0.05).

**Figure 3:**
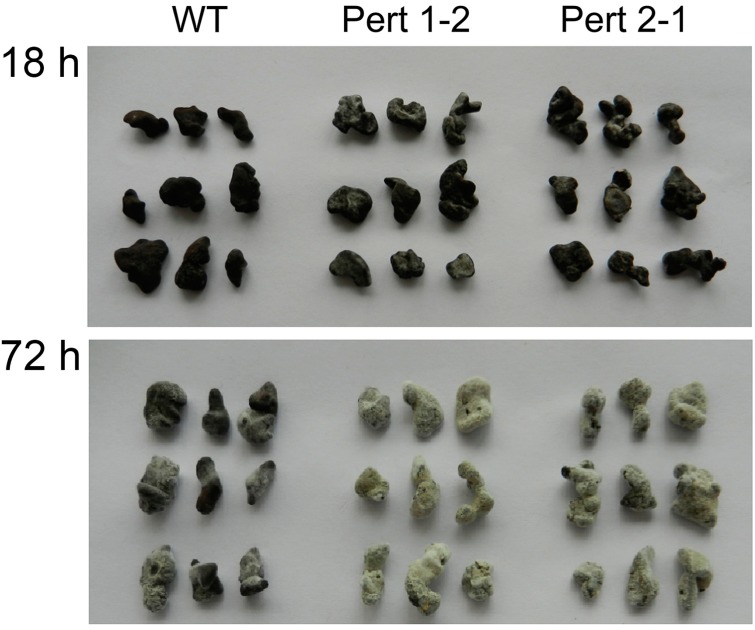
Parasitism of *C. rosea* f. *catenulata* HL-1-1 and *Per3* gene transformants on sclerotia of *S. sclerotiorum* at different stages. Note: WT: Wild type.

**Figure 4:**
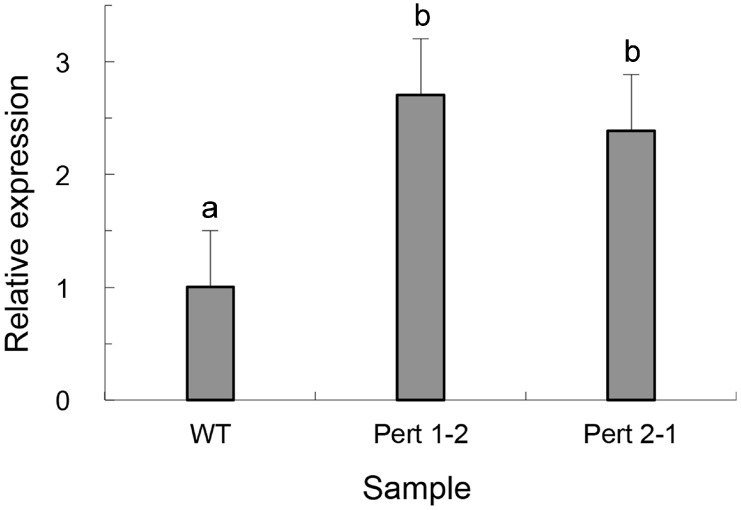
Expression of *Per3* in HL-1-1 transformants as assessed by quantitative real-time PCR. Note: WT: Wild type. Error bars indicate the standard deviation of three replicates. Different letters represent significant differences (*p <* 0.05) according to Duncan’s multiple range test.

Compared with the wild type, the mutants showed different parasitic abilities on the sclerotia of *S. sclerotiorum* after overexpression of *Per3*, and time after infection at which the fungal mycelia appeared on the sclerotia differed from 9 to 15 h. Among the transformants, Pert 1-2 and Pert 2-1 showed remarkably strong parasitic abilities. Parasitism of both isolates could be observed after 9 h and their parasitic rates were more than 95% at 12 h, while only 36.7% of the sclerotia were parasitized by the wild strain at 12 h (Table 2). After three days, the surfaces of the sclerotia infected by the mutants were covered with hyphae and spores of the mycoparasites, and the whole sclerotia turned to soft rot. For the wild type, the hyphae were extended and relatively loose and the sclerotia remained relatively firm, indicating that the parasitism of the *Per3* overexpressing transformants was enhanced compared with the wild type (Figure 3). Real-time PCR showed that the expression levels of *Per3* in transformants Pert 1-2 and Pert 2-1 were more than twice that of the wild type (Figure 4), indicating that the increased expression level of *Per3* promoted mycoparasitism of *C. rosea* f. *catenulate* HL-1-1.

**Table 2:** Parasitism of *C. rosea* f. *catenulata* HL-1-1 and *Per3* gene transformants on the sclerotia of *S. sclerotiorum*.

Strain	Parasitic Rate (%)			
	9 h	12 h	15 h	18 h
HL-1-1	3.3 ± 0.0 ^a^	36.7 ± 0.4 ^a^	86.7 ± 0.8 ^a^	100.0 ± 0.0 ^a^
Pert 1-2	30.0 ± 0.4 ^b^	96.7 ± 0.7 ^b^	100.0 ± 0.0 ^b^	100.0 ± 0.0 ^a^
Pert 2-1	26.7 ± 0.2 ^c^	100.0 ± 0.0 ^c^	100.0 ± 0.0 ^b^	100.0 ± 0.0 ^a^

Different letters in the same column represent significant differences (*p <* 0.05) according to Duncan’s multiple range test.

## 3. Discussion

For plant disease management, it is of great value to develop biocontrol-related genes. In this study, 502 clones from the SSH cDNA library of *C. rosea* f. *catenulata* HL-1-1 were sequenced, and some ESTs exhibited homology to a wide range of genes whose protein products were associated with the destruction of cell-walls and prevention of the growth of fungal pathogens, e.g., glucanase, ribosomal protein and cytochrome P450. These genes had been identified in mycoparasites, pathogenic fungi and plants under fungal stress [24,26,27,29]. Furthermore, a great number of ESTs with unknown function and potential new genes were detected in the differentially expressed cDNA library of *C. rosea* f. *catenulata*.

In many cases, mycoparasites lived by saprotrophy. However, a few studies suggested that some species possess both saprotrophic and biotrophic lifestyles [30]. Mycoparasites perform their biotrophic penetration using various parasitic structures, such as appressorium. Dugan *et al.* found that appressoria formed in *C. rosea* when parasitizing on hypha of *Didymella rabiei* [31]. Chatterton and Punja detected an appressorium-like structure from *C. rosea* f. *catenulate* when it attached to the hyphae of *P. aphanidermatum* [4]. *Trichoderma* spp. could also form appressorium-like structures in the process of parasitizing the hyphae of *R. solani* [32].

A perilipin factor was reported to affect the turgor pressure of appressoria via regulating lipid droplet metabolism, and further weaken the penetration to insect cuticula [21]. Disruption of the perilipin encoding gene, *Mpl1*, resulted in an impaired ability to store lipid, and the appressoria of the mutant lacked lipid droplets compared with the wild type, leading to a dramatic reduction of turgor pressure and mechanical penetration to host cuticles. In our study, we selected a perilipin-like protein encoding gene, *Per3*, whose expression level was significantly up-regulated among these functional ESTs. Real-time quantitative PCR of *Per3* showed that its expression was greatly up-regulated under induction by *S. sclerotiorum*, indicating that perilipin might be associated with the formation of turgor pressure in appressoria and in the process of parasitization on *S. sclerotiorum*. At the early stage of sclerotia induction, the expression level declined, which might reflect the lack of nutrition in the sclerotia powder medium, such that *C. rosea* f. *catenulata* HL-1-1 had to decompose lipid droplets through its lipid metabolic pathway to germinate and grow when it was inoculated into a nutrient-deficient broth. Accordingly, the expression level of *Per3*, whose protein product bound with lipid droplets, was reduced. After a period of time when HL-1-1 could obtain nutrients by decomposing the sclerotia powder, the fungus changed to storing lipid droplets instead of degrading them, and the expression level of *Per3* began to increase. In the overexpressing transformants, higher expression of *Per3* might have a greater effect on turgor pressure formation in the appressoria, which might further improve penetration of the mycoparasites.

As a member of the perilipin, adipocyte differentiation-related protein and TIP47 (PAT) family proteins, perilipin affects lipid droplet metabolism of organisms and plays a crucial role in lipid droplet motion, which is conducive to lipid homeostasis [17]. Previous studies indicated that various factors could affect the expression of perilipin genes [33,34,35]. In our study, when *Clonostachys rosea* f. *catenulata* HL-1-1 was induced by sclerotia powder, the expression of perilipin changed remarkably. The selective expression of *Per3* suggested that variation of the expression of perilipin protein could modulate the hydrolysis of adipocytes, and transform the states of lipid droplets between dispersion and cluster in the fungal cytoplasm, which is consistent with reports in mouse fibroblasts and *Drosophila* embryos [18,36].

The functional verification of *Per3* by overexpressing the gene in *C. rosea* f. *catenulata* HL-1-1 during its interaction with *S. sclerotiorum* indicated that *Per3* was involved in mycoparasitism. To the best of our knowledge, this is the first report of the involvement of a perilipin-like gene in mycoparasitism of *C. rosea* f. *catenulata*. Further study of perilipin in *Clonostachys rosea* f. *catenulata* might lead to novel methods to combat fungal plant pathogens.

## 4. Experimental Section

### 4.1. Strains

*C. rosea* f. *catenulata* HL-1-1 was isolated from a vegetable plantation in Hunan Province, and *S. sclerotiorum* Ss-H was separated from soybean Sclerotinia stems in field in Heilongjiang Province. Both strains are maintained in the Biocontrol of Soilborne Diseases Lab of the Institute of Plant Protection, Chinese Academy of Agricultural Sciences (CAAS).

### 4.2. Preparation of Sclerotia Powder Medium

A slope culture of *S. sclerotiorum* Ss-H was inoculated onto potato dextrose agar (PDA) plate andincubated at 26 °C for 7 days. The agar was cut into approximately 10 mm disks using a sterile puncher and inoculated into carrot medium [37] at 10–15 blocks per flask. After incubation at 26 °C for 15–20 days, sclerotia were formed in the carrot media. The remnants of the carrot media were discarded by rinsing with tap water several times, and the sclerotia were harvested. The sclerotia were then ground into 1–2 mm granules using a high-speed grinder (Hongguang Industry and Trade, Jiangsu, China). Liquid water containing 2% sclerotia powder was used as sclerotia powder broth to induce gene expression in HL-1-1.

### 4.3. DNA Sequencing and Analyzing

A total of 502 clones from the cDNA library constructed previously were selected for sequencing using the universal primer M13 on an ABI 3730XL DNA sequencer (Applied Biosystems, Foster City, CA, USA). The ESTs obtained were analyzed using Phred software (University of Washington, Seattle, WA, USA) [38]. ESTs less than 100 bp were discarded. Clustering of ESTs into unigenes was performed using the SeqMan program (DNAStar Inc., Madison, WI, USA).

Unigenes were compared with entries in the non-redundant (nr) and EST databases in GenBank using BlastX and BlastN (http://www.ncbi.nlm.nih.gov). Matches with an E-value below 10^−5^ were considered to provide biologically meaningful information. The MIPS database (http://mips.helmholtzmuenchen.de/proj/fun catDB) was used to identify the functional categories of the ESTs, and Blast2GO software (BioBam Bioinformatics S.L., Valencia, Spain) was used to obtain the GO annotations regarding biological process, molecular function and cellular components associated with the ESTs.

### 4.4. RNA Extraction and cDNA Synthesis

HL-1-1 was cultured in PD broth on a shaking table at 180 r·min^−1^. After growing for 3 days at 28 °C, the fermentation broth was filtered through a 25-μm pore size sieve and washed five times with sterile distilled water to collect mycelia. One milliliter of mycelia suspension was inoculated into 100 mL sclerotia powder broth in a 500 mL flask, and cultured at 28 °C on a shaking table at 180 r·min^−1^. The mycelia of HL-1-1 were collected at 0, 6, 12, 24, 48, 72, and 96 h, frozen immediately in liquid nitrogen and homogenized with sterile mortars and pestles. Total RNA was extracted using Trizol reagent (Invitrogen, Carlsbad, CA, USA) according to the manufacturer’s protocol, and DNase I (Invitrogen, Carlsbad, CA, USA) was used to remove DNA contamination. First strand cDNA was synthesized using a cDNA Synthesis Kit (TaKaRa, Dalian, China) and kept at −80 °C.

### 4.5. Real-Time PCR

Expression of perilipin-related gene *Per3* was quantified by real-time PCR, in which β*-tubulin* was used as an internal reference gene [39]. Primer pairs for both genes were designed using the software Primer Premier 5.0 (Permier Biosoft, Palo Alto, CA, USA) (Table 3). The specificity of the primers was certified by conventional PCR with the following program: 94 °C for 3 min; 30 cycles of 94 °C for 1 min, 55 °C for 30 s and 72 °C for 30 s; followed by 72 °C for 10 min.

**Table 3:** Primers used in the assay.

Primers	Sequence (5'-3')	Purpose
SPF	CGTTGTCAAGAAGCCTACCG	Real-time PCR
SPR	GAGGCCCTTCTGCTCAATCT	
β*-tubulin* F	CATCTTCAGACCGGTCAGTG	Real-time PCR
β*-tubulin* R	AAGTAGACGTTCATGCGCTC	
3'OPF	AGATTGAGCAGAAGGGCCTC	3'RACE
3'OPR	TCAACCAGTAAGGCGAGAAT	
5'OPF	AGTGAGGAAGCTGCTAATCC	5'RACE
5'OPR	ATCTTCTTGATCTCGCTCGA	
*Per3* F	GAAACTTCTCTTTCGTCTCTATCGA	Amplification of complete DNA and full-length cDNA
*Per3* R	CTTGCAAGCACAGAAAGAAAATCAA	
*qdz*F	GAATTCCCTTGTATCTCTA	*qdz* amplification
*qdz*R	AAGAGAAAAGAAAAGAGCA	
*zzz*F	CCGACCGGGGATCCACTTA	*zzz* amplification
*zzz*R	GGAGTGGGCGCTTACACAG	

The samples were diluted 10-fold, and the expression level of *Per3* was determined using a SYBR Premix Ex Taq (TaKaRa, Dalian, China) on an IQ 5™ multicolor real-time PCR detection system (Bio-Rad, Hercules, CA, USA). The 25 μL reaction system contained 12.5 μL of SYBR Premix, 2 μL of diluted cDNA, 1 μL of each primer, and 8.5 μL of RNase-free water. Real-time PCR was performed in a 96-well plate with the following program: 95 °C for 2 min; followed by 40 cycles of 95 °C 10 s, 55 °C 20 s and 72 °C 30 s. After the reaction, fluorescence values were collected every 0.5 °C from 55 to 85 °C for 81 cycles to check for non-specific amplification. The relative expression level of *Per3* was calculated using 2^−ΔΔ*C*t^ method [40]. Three replicates were performed for each template at each time point.

### 4.6. Cloning of Full-Length cDNA of the Per3 Gene

The full-length cDNA of *Per3* was acquired using the 5'-Full RACE Kit and 3'-Full RACE Core Set Ver. 2.0 (TaKaRa, Dalian, China), following the manufacturer’s instructions. Nested PCR was carried out using LA-Taq (TaKaRa, Dalian, China) as follows: 94 °C for 3 min; 30 cycles of 94 °C for 1 min, 55 °C for 30 s and 72 °C for 1 min; followed by 72 °C for 10 min. The amplified products were recovered from agarose gel using Agarose Gel DNA Fragment Recovery Kit Ver. 2.0 (TaKaRa, Dalian, China) and cloned into vector pMD19-T (TaKaRa, Dalian, China) for sequencing. By assembling products of 3' and 5'RACE, the full-length cDNA sequence of gene *Per3* was acquired, and its validity was verified by PCR using primers 3'OPF, 3'OPR, 5'OPF and 5'OPR (Table 3), and the following program: 94 °C for 4 min; 30 cycles of 94 °C for 30 s, 55 °C for 1 min, and 72 °C for 1.5 min; and 72 °C for 10 min as the final extension. The genomic DNA was extracted using a Fungal DNA Mini Kit (OmegaBio-tek, Doraville, GA, USA), and the complete DNA sequence of *Per3* was amplified with the same primers and conditions as above.

### 4.7. Bioinformatic Analysis of the Full-Length cDNA of Per3

The full-length cDNA of *Per3* was analyzed and compared using Blast (http://www.ncbi.nlm.nih.gov/blast/), and the ORF was predicted at ORF Finder (http://www.ncbi.nlm.nih.gov/gorf/gorf.html). The molecular weight and isoelectric point of the predicted protein were calculated by Compute pI program [41], its hydrophobicity profile was investigated by ProtScale program [42], and signal peptide prediction was conducted by Signal 4.1 program [43]. Transmembrane regions of Per3 were predicted using TMPRED software (Memorec Stoffel, Koeln, Germany) [44], and functional domains were analyzed using the Conserved Domain Database V3.10 (hppt://www.ncbi.nlm.nih.gov/Structure/cdd/cdd.shtml). Per3 was compared with similar sequences from other species, which were obtained from GenBank using Blast and aligned using DNAMAN 6.0 software (Lynnon Biosoft, Quebec, QC, Canada). Phylogenetic analysis was performed using MEGA version 5.1 with 1000 bootstraps [45], and evolutionary distances among the species were calculated using the neighbor joining method [46].

### 4.8. Plasmid Construction and Transformation

The plasmid pAN7-1, containing the promoter of the *gpdA* gene (glyceraldehydes-3-phosphate dehydrogenase), a termination region from *trpC* of *Aspergillus nidulans* [47], and a hygromycin B resistance gene *hph*, was used to construct an expression vector for HL-1-1. Fragments of *gpdA* and *trpC* were amplified by using PCR with primers of *qdz*F, *qdz*R and *zzz*F, *zzz*R, respectively (Table 3). Plasmid pAN7-1 was digested with *HindIII* (TAKARA, Dalian, China) and ligated to insert the DNA of *Per3* together with *gpdA* and *trpC*. Plasmid pAN7-1-*Per3* was then transformed into isolate HL-1-1 by the protoplast transformation method [48]. The transformants were transferred on PDA for three generations and selected by hygromycin resistance, as described previously [48].

### 4.9. Growth and Sporulation of the Transformants

One hundred and two genetically stable transformants were selected randomly to determine their growth and sporulation in a Petri dish (9 cm). After growing on PDA at 26 °С for 1 week, the colony diameters were measured and the numbers of spores were counted by eluting spores from the hyphae with 10 mL sterile distilled water and a glass spatula. Three replicates were conducted for each transformant.

### 4.10. Mycoparasitism of the Transformants on Sclerotia of S. sclerotiorum

*S. sclerotiorum* Ss-H was cultured in carrot medium at 26 °С for 10 days. The sclerotia were harvested and the medium and hyphae of Ss-H were removed by washing with sterile distilled water. A sufficient quantity of sclerotia was surface-sterilized in 75% ethanol for 45 s, 2.5% sodium hypochlorite solution for 3 min, and 75% ethanol for 45 s, followed by rinsing with sterile distilled water five times. The sclerotia were immersed in spore suspensions of all transformants at a concentration of 1 × 10^7^ spores·mL^−1^ for 10 min and then the excess water was sucked out. Twenty uniform sclerotia were placed on a wet sterile filter paper and incubated at 26 °С. The number of sclerotia parasitized by the fungal isolates was counted under an inverted microscope (BX41, Olympus, Tokyo, Japan) and a stereo-microscope (SMZ-10, Nikon, Tokyo, Japan) at 9, 12, 15, and 18 h. Mycelia of the transformants extending on the surface of sclerotia were regarded as parasitic, and the wild type HL-1-1 was treated as the control. Three replicates were used for each transformant.

### 4.11. Transcription Level of Per3 in the Transformants

Total RNA and cDNA of the transformants with high parasitic ability on sclerotia were extracted and synthetized by using Trizol reagent (Invitrogen, Carlsbad, CA, USA) and a cDNA Synthesis Kit (TaKaRa, Dalian, China), respectively. Expression levels of *Per3* in the transformants were quantified by real-time PCR using the primers of SPF and SPR (Table 3), using the same procedure described above. Three replicates were performed.

### 4.12. Statistical Analysis

The statistical software SAS 9.1.3 (SAS Institute Inc., Cary, NC, USA) was used for analysis of variance (ANOVA). Duncan’s multiple range test was used to compare the means from each treatment. *p*-values < 0.05 were considered significant.

## 5. Conclusions

Our study identified a perilipin protein encoding gene *Per3*, which was obtained from a SSH cDNA library of *C. rosea* f. *catenulata* HL-1-1 parasitizing sclerotia of *S*. *sclerotiorum*. *Per3* was up-regulated under the induction of sclerotia, and overexpression of *Per3* significantly enhanced parasitic ability on sclerotia compared with the wild type. This study indicated that *Per3* might be involved in the mycoparasitic process of *C. rosea* f. *catenulata* HL-1-1.

## Supplementary Materials

Supplementary materials can be found at http://www.mdpi.com/1422-0067/16/03/5347/s1.
