# Supplementary material for: A Perilipin Gene from Clonostachys rosea f. Catenulata HL-1-1 Is Related to Sclerotial Parasitism
Source: Int J Mol Sci. 2015 Mar 9;16(3):5347–62. doi: 10.3390/ijms16035347 (PMC4394479; doi:10.3390/ijms16035347)
Supplement: Supplementary file 1 [file ijms-16-05347-s001.pdf]

Supplementary Information

Table S1. Gene Ontology functional annotation of differently expressed genes.

Gene	Biological Process P	Molecular Function F	Cellular Component
2-25	Phosphate ion transport	Inorganic phosphate transmembrane transporter activity	Membrane
2-26	Cellular carbohydrate metabolic process	Transferase activity; Transferring acyl groups	-
2-49	-	Oxidoreductase activity; Binding	-
2-90	Oxidation-reduction process	Oxidoreductase activity; Iron ion binding; Heme binding	-
3-4	-	Phosphopantetheine binding	-
4-33	Aromatic amino acid family metabolic process	4-hydroxyphenylpyruvate dioxygenase activity	-
4-37	-	Protein binding	-
4-47	Transmembrane transport	Transporter activity	Mitochondrial inner membrane; Integral to membrane
7-13	-	-	Integral to membrane
7-32	-	Protein binding	-
7-73	Oxidation-reduction process	Oxidoreductase activity	-
8-7	ATP synthesis coupled proton transport	Hydrogen ion transmembrane transporter activity	Mitochondrial proton-transporting ATP synthase complex-coupling factor
8-20	Carbohydrate metabolic process	Hydrolase activity; Hydrolyzing *O*-glycosyl compounds	-
8-24	Chitin biosynthetic process	Chitin synthase activity	-
8-70	ER to Golgi vesicle-mediated transport	-	Intracellular
8-76	Protein folding; Replicative cell aging	-	Mitochondrial inner membrane
8-89	Oxidation-reduction process	Oxidoreductase activity; Molybdenum ion binding	-
9-25	Translation	Structural constituent of ribosome	Ribosome

"-" means no annotation in this term. Genes without annotation in all three terms were not listed.

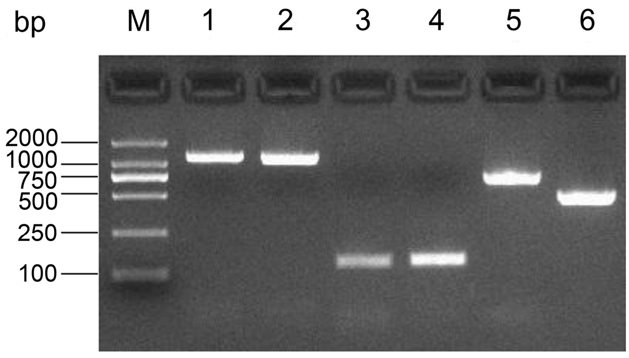

Figure S1. Agarose gel electrophoresis. M: Marker; Lane 1: Complete DNA of *Per3*; Lane 2: Full-length cDNA of *Per3*; Lane 3: PCR amplification of β-*tubulin* using specific primers β-*tubulin* F and β-*tubulin* R; Lane 4: PCR amplification of *Per3* using primers *Per3* F and *Per3* R; Lane 5: 5′RACE of *Per3*; Lane 6: 3′RACE of *Per3*.
